# A new nodosaurid ankylosaur (Dinosauria: Thyreophora) from the Upper Cretaceous Menefee Formation of New Mexico

**DOI:** 10.7717/peerj.5435

**Published:** 2018-08-24

**Authors:** Andrew T. McDonald, Douglas G. Wolfe

**Affiliations:** 1 Western Science Center, Hemet, CA, USA; 2 Zuni Dinosaur Institute for Geosciences, Springerville, AZ, USA

**Keywords:** *Invictarx zephyri*, Nodosauridae, Ankylosauria, Allison Member, Menefee Formation

## Abstract

Nodosauridae is a clade of armored dinosaurs with a rich fossil record and long history of study in North America. Nodosaurid fossils have been collected throughout the western United States and Canada. Here, we report three new nodosaurid specimens from the Upper Cretaceous (lower Campanian) Allison Member of the Menefee Formation, San Juan Basin, northwestern New Mexico. The three specimens belong to a new genus and species, *Invictarx zephyri*, characterized by a unique combination of features pertaining to the morphology of the osteoderms. Among the three specimens there are representative cervical/pectoral and thoracic osteoderms, as well as components of a probable co-ossified pelvic shield. The new tax on is most similar to *Glyptodontopelta mimus* from the Maastrichtian of New Mexico.

## Introduction

Ankylosauria is a clade of ornithischian dinosaurs that are armored with a diverse array of osteoderms and that have a rich fossil record from the Cretaceous of North America (Carpenter & Kirkland, 1998; Carpenter et al., 1999; Vickaryous, Maryańska & Weishampel, 2004; Arbour & Currie, 2013; Arbour, Zanno & Gates, 2016). Herein we describe three isolated, incomplete postcranial skeletons of a new genus and species of nodosaurid ankylosaur from the Allison Member of the Upper Cretaceous Menefee Formation. This new taxon is distinguished from other nodosaurids by a unique combination of characters, and is the first dinosaur diagnostic to species reported from the Menefee Formation.

The Menefee Formation forms an expanse of badlands throughout the San Juan Basin of northwestern New Mexico. A precise temporal framework for the entire Menefee is not yet available. Lucas et al. (2005) obtained an Ar/Ar radioisotopic date of 78.22 ± 0.26 Ma from a bentonite layer near the top of the formation in the Gallina hogback in the eastern part of the San Juan Basin. In the part of the San Juan Basin where the nodosaurid fossils were collected, the overlying marginal marine Cliff House Sandstone contains fossils of the ammonoid *Baculites perplexus* (Siemers & King, 1974), corresponding to an age of between 78.0 and 78.5 Ma (Molenaar et al., 2002). According to the regional stratigraphic correlation chart of Molenaar et al. (2002), the Menefee Formation spans approximately 84.0–78.5 Ma, based upon correlations with marine biostratigraphic zones. This age range corresponds to uppermost Santonian—middle Campanian (Cohen et al., 2013).

**Figure 1:**
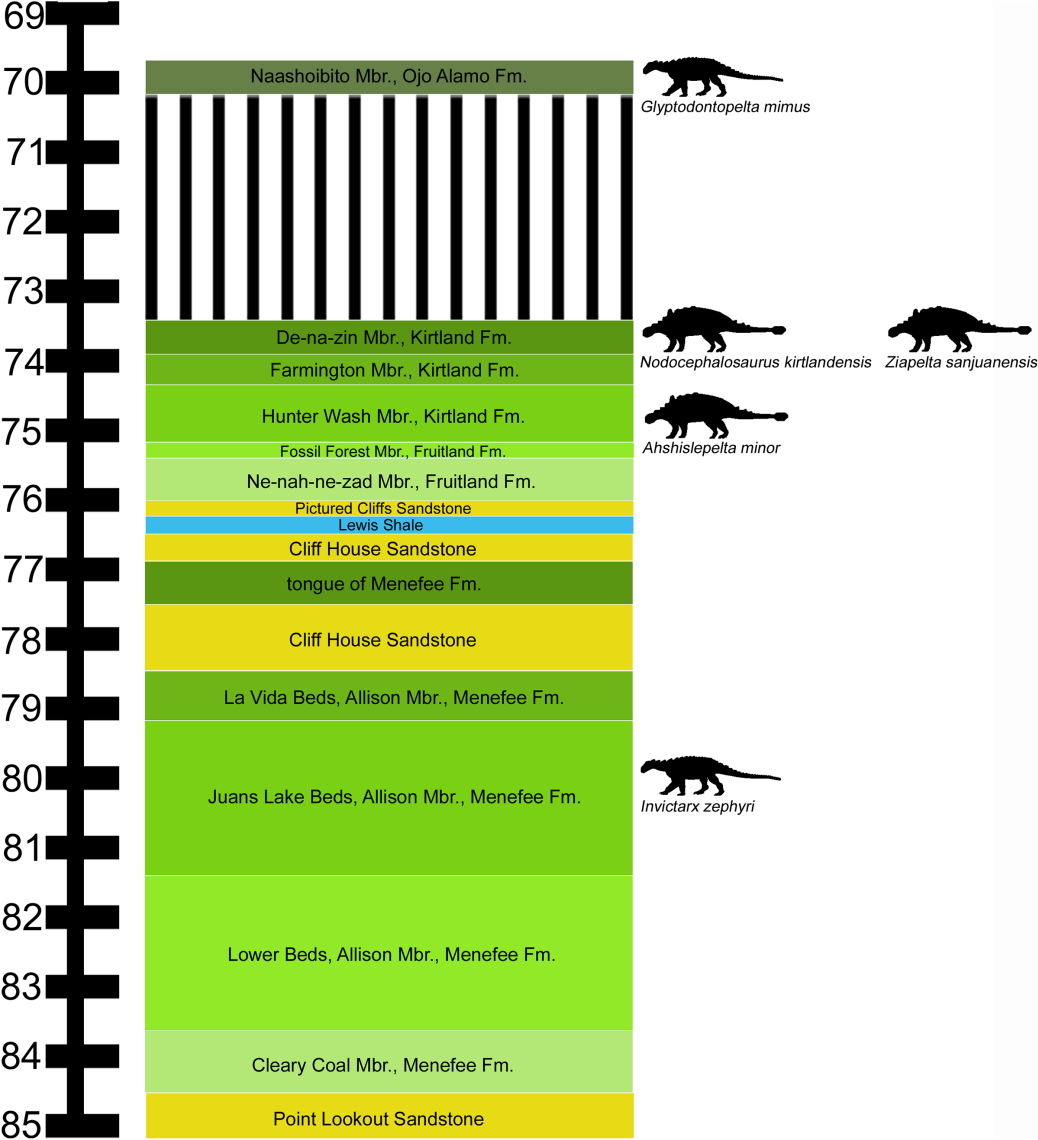
Stratigraphic occurrences of *Invictarx zephyri* and other ankylosaurs from the San Juan Basin. Generalized stratigraphic column of Upper Cretaceous strata in the San Juan Basin, northwestern New Mexico, showing the stratigraphic positions of the nodosaurids *I. zephyri* and *Glyptodontopelta mimus* and the ankylosaurids *Ahshislepelta minor*, *Nodocephalosaurus kirtlandensis*, and *Ziapelta sanjuanensis*. Ankylosaur occurrence data are from Sullivan & Lucas (2015). Nodosaurid silhouette by Scott Hartman (https://creativecommons.org/publicdomain/zero/1.0/), and ankylosaurid silhouette by Andrew A. Farke (https://creativecommons.org/licenses/by/3.0/), both available from PhyloPic. Stratigraphic column is derived from data in Miller, Carey & Thompson-Rizer (1991), Molenaar et al. (2002), Sullivan & Lucas (2006), and Fowler (2017).

We follow the stratigraphic nomenclature of the Menefee Formation proposed by Miller, Carey & Thompson-Rizer (1991), who described the detailed geology in the vicinity of our field area. The Menefee Formation is divided into two members, the Cleary Coal and Allison. Miller, Carey & Thompson-Rizer (1991) further divided the Allison Member into the Lower Beds, Juans Lake Beds, and La Vida Beds (Fig. 1). Vertebrate fossils have been reported from the Menefee, primarily from the Allison Member, but these are mostly fragmentary (Hunt & Lucas, 1993). Exceptions include the associated skeleton of an indeterminate centrosaurine ceratopsid (Williamson, 1997) and the holotype skull of the basal alligatoroid *Brachychampsa sealeyi* (Williamson, 1996) (considered a subjective junior synonym of *Brachychampsa montana* by Sullivan & Lucas (2003)).

In 2011, ATM and DGW initiated a project to explore outcrops of the Allison Member for new vertebrate localities, supported by the University of Pennsylvania and volunteers from the Southwest Paleontological Society, and later by the Western Science Center (WSC) and Zuni Dinosaur Institute for Geosciences. The field area consists predominantly of exposures of fluvial mudstones and sandstones in the Juans Lake Beds (Miller, Carey & Thompson-Rizer, 1991). Discoveries to date have added greatly to the vertebrate record from the Allison Member, including material of three types of turtles; neosuchians distinct from *Brachychampsa sealeyi*; and hadrosaurid, ceratopsid, and theropod dinosaurs. The additional dinosaurs and other vertebrates we have discovered in the Allison Member will be described in a series of forthcoming publications.

## Materials and Methods

The specimens described herein were collected under the following permits issued by the U.S. Bureau of Land Management (BLM): NM11-005S, NM12-03S, and NM16-11S.

The electronic version of this article in portable document format will represent a published work according to the International Commission on Zoological Nomenclature (ICZN), and hence the new names contained in the electronic version are effectively published under that Code from the electronic edition alone. This published work and the nomenclatural acts it contains have been registered in ZooBank, the online registration system for the ICZN. The ZooBank Life Science Identifiers (LSIDs) can be resolved and the associated information viewed through any standard web browser by appending the LSID to the prefix http://zoobank.org/. The LSID for this publication is urn:lsid:zoobank.org:pub:0E04AA40-BEA4-4A22-9CCB-6ACE419607B5. The online version of this work is archived and available from the following digital repositories: PeerJ, PubMed Central, and CLOCKSS.

## Results

### Systematic paleontology

Dinosauria Owen, 1842, sensu Baron, Norman & Barrett, 2017
Ornithischia Seeley, 1888, sensu Sereno, 2005
Thyreophora Nopcsa, 1915, sensu Sereno, 2005
Ankylosauria Osborn, 1923, sensu Sereno, 2005
Nodosauridae Marsh, 1890, sensu Sereno, 2005
*Invictarx zephyri* gen. et sp. nov.

Holotype: WSC 16505, incomplete postcranial skeleton including fragments of a dorsal rib, six complete or partial identifiable osteoderms (WSC 16505.1–WSC 16505.6), and fragments of additional osteoderms.

Referred specimens: Natural History Museum of Utah (UMNH) VP 28350, incomplete postcranial skeleton including three dorsal vertebrae, fragments of dorsal ribs, distal end of left humerus, distal end of left ulna, proximal ends of left and right radii, incomplete metacarpal, numerous incomplete but identifiable osteoderms, and fragments of additional osteoderms; UMNH VP 28351, incomplete postcranial skeleton including fragments of several dorsal centra, fragments of dorsal ribs, numerous incomplete but identifiable osteoderms, and fragments of additional osteoderms.

Etymology: *Invictarx* is derived from the Latin words invictus (“invincible, unconquerable”) and *arx* (“fortress”), in reference to the well-armored nature of ankylosaurian dinosaurs. The specific name, *zephyri*, is the genitive form of the Latin masculine noun *zephyrus*, “west wind,” in reference to the blustery conditions that prevail among the outcrops where the specimens were discovered. The full name may be translated as “unconquerable fortress of the western wind.”

Locality: All specimens were collected in San Juan County, New Mexico, on land administered by the U.S. BLM. Precise locality data are on file at WSC, UMNH, and the BLM.

Horizon: All specimens were collected from outcrops of the Juans Lake Beds (Miller, Carey & Thompson-Rizer, 1991) (Fig. 1), upper part of the Allison Member, Menefee Formation; lower Campanian, Upper Cretaceous (Molenaar et al., 2002; Lucas et al., 2005).

Specific diagnosis (as for genus by monotypy): nodosaurid ankylosaur distinguished by the following unique combination of characters: (1) observable on WSC 16505, UMNH VP 28350, and UMNH VP 28351 cervical/pectoral, thoracic, and pelvic osteoderms exhibit overall smooth surface texture, with little or no projecting rugosity, with abundant pits distributed randomly over the entire external surface, and with no neurovascular grooves or a small number of bifurcating and non-bifurcating neurovascular grooves distributed randomly, similar to *Glyptodontopelta mimus* but lacking the dense pattern of dendritic grooves that characterizes that taxon (Burns, 2008; Burns & Currie, 2014); (2) observable on WSC 16505 and UMNH VP 28351 some thoracic osteoderms exhibit a low, rounded keel with a deep groove extending craniocaudally along the apex, also present in the ankylosaurids *Anodontosaurus lambei* (Fig. 13G in Penkalski (2018)) and *Platypelta coombsi* (Fig. 13O in Penkalski (2018)) (P. Penkalski, 2018, personal communication), but absent in *G. mimus* (Burns, 2008); and (3) observable on UMNH VP 28351 probably possessed a co-ossified pelvic shield consisting of polygonal osteoderms of uniform size (Category 3 of Arbour, Burns & Currie (2011)), similar to some other nodosaurids, including *Nodosaurus textilis* (Lull, 1921), *Stegopelta landerensis* (Moodie, 1910), *G. mimus* (Ford, 2000; Burns, 2008), and *Europelta carbonensis* (Kirkland et al., 2013), as well as the ankylosaurid *Aletopelta coombsi* (Ford & Kirkland, 2001; Arbour & Currie, 2016).

### Description

The holotype and both referred specimens represent nodosaurids, as indicated by the thick postcranial osteoderms with flat or slightly concave basal surfaces (Coombs, 1978; Carpenter, 2001). Furthermore, the referred specimen UMNH VP 28350 is identifiable as a nodosaurid based upon the circular shape of the proximal articulation surface of the right radius (see below) (Coombs, 1978; Vickaryous, Maryańska & Weishampel, 2004). Therefore, comparisons will focus primarily on other members of Nodosauridae. It can be difficult to determine the precise life positions of disarticulated postcranial osteoderms, such as those present in the three specimens of *I. zephyri*. Examples of in situ osteoderms are known for some nodosaurids, including *Sauropelta edwardsorum* (American Museum of Natural History (AMNH) 3036; Ostrom, 1970; Carpenter, 1984), *Borealopelta markmitchelli* (Tyrrell Museum of Palaeontology (TMP) 2011.033.0001; Brown et al., 2017), and *Edmontonia rugosidens* (AMNH 5665; Gilmore, 1930). However, these taxa are quite dissimilar from each other in osteoderm morphology and arrangement (see Figs. 3C–3E in Brown et al. (2017)), demonstrating that different nodosaurid taxa probably varied greatly in their overall appearances. Despite this caveat, there are some useful points of resemblance with the articulated cervical/pectoral half-rings of *E. rugosidens* (AMNH 5665) (see cervical/pectoral osteoderms of WSC 16505 below). For the purposes of this description, it is assumed that *I. zephyri* possessed three cervical/pectoral half-rings, as is typical for nodosaurids, including *Edmontonia rugosidens* (AMNH 5665; Gilmore, 1930), *S. edwardsorum* (AMNH 3035; Carpenter & Kirkland, 1998), *S. condrayi* (Eaton, 1960; Carpenter & Kirkland, 1998), *Borealopelta markmitchelli* (Brown et al., 2017), and *Struthiosaurus austriacus* (Pereda Suberbiola & Galton, 2001).

**Figure 2:**
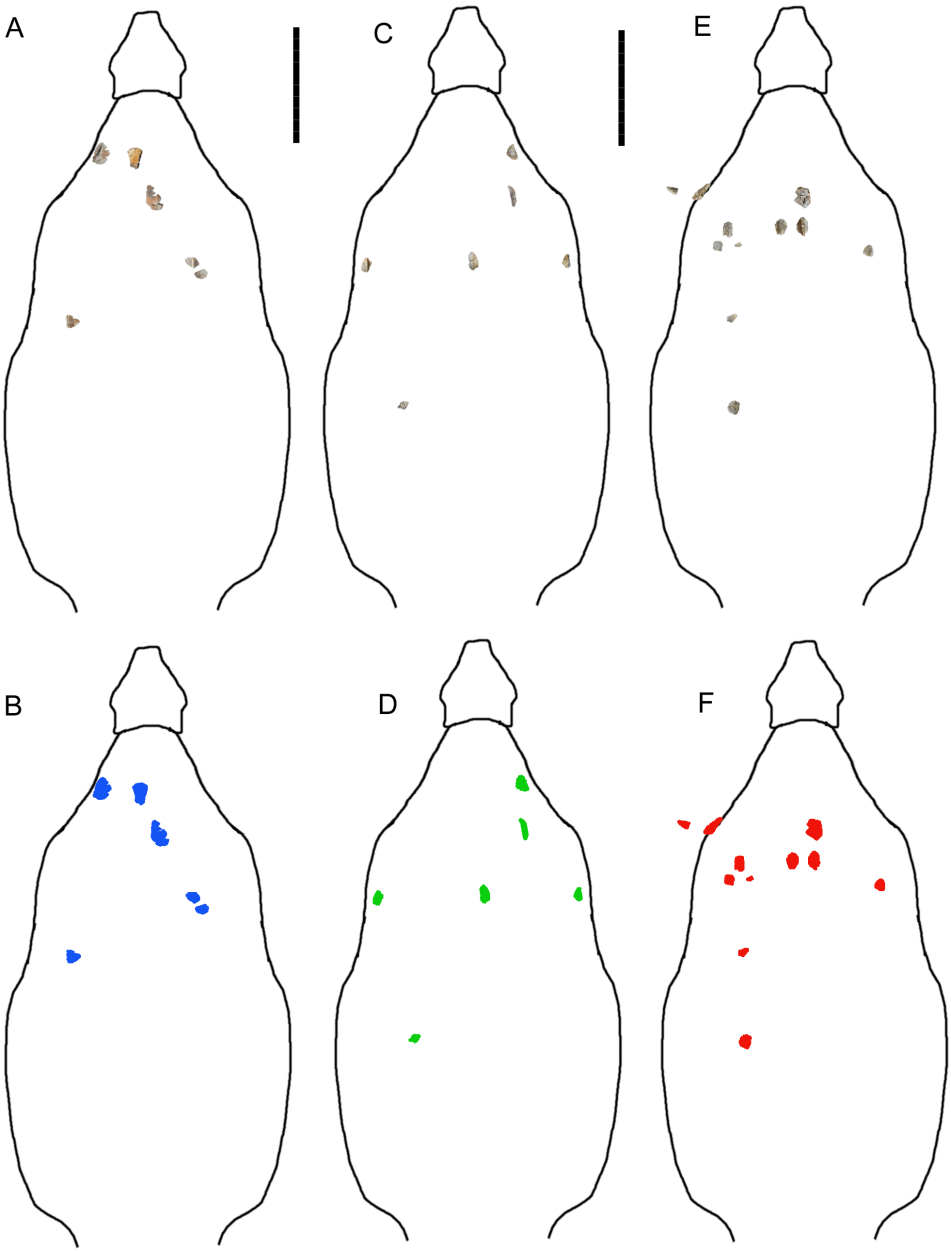
Reconstructions of identifiable osteoderm placements in the three specimens of *I. zephyri*. WSC 16505 (holotype) osteoderms (A) and color-code (B). UMNH VP 28350 osteoderms (C) and color-code (D). UMNH VP 28351 osteoderms (E) and color-code (F). All osteoderms are set to the same scale. Body outlines based upon skeletal reconstruction of *S. edwardsorum* (Fig. 2 in Carpenter (1984)). Scale bars equal 50 cm.

Further insight may be gained from comparisons with *G. mimus* (Ford, 2000), which includes a referred specimen, State Museum of Pennsylvania (SMP) VP-1580, with 71 complete or nearly complete osteoderms from the cervical/pectoral, thoracic, and pelvic regions (Burns, 2008). *Invictarx* and *Glyptodontopelta* both are known exclusively from the San Juan Basin of northwestern New Mexico; *Invictarx* probably possessed a co-ossified pelvic shield similar to that of *Glyptodontopelta*; and many of the osteoderms available for *Invictarx* conform to the seven osteoderm morphotypes, A–G, identified in *Glyptodontopelta* by Burns (2008). However, it should be noted that these taxa have been found in units (Juans Lake Beds, Allison Member, Menefee Formation and Naashoibito Member, Ojo Alamo Formation, respectively) separated by roughly 10 million years (Molenaar et al., 2002; Lucas et al., 2005; Jasinski, Sullivan & Lucas, 2011) (Fig. 1), and differ in osteoderm morphology. The inferred placements of the osteoderms of *Invictarx* and reconstructions of its appearance could well change with additional discoveries (Fig. 2).

Quarry maps are not available for the three specimens. All the material was collected as localized float that had eroded onto the surface. Test excavations and subsequent monitoring of the sites have not revealed additional bones. In the following descriptions, osteoderm positional, morphological, and directional terminology follows Penkalski (2001) and Burns & Currie (2014). Digital 3D models of WSC 16505, UMNH VP 28350, and UMNH VP 28351 are available at MorphoSource under the project name “A new nodosaurid ankylosaur from the Upper Cretaceous Menefee Formation of New Mexico.”

#### Description of WSC 16505 (holotype)

Western Science Center 16505 consists of small fragments of a dorsal rib, six complete or partial identifiable osteoderms (WSC 16505.1–WSC 16505.6), and fragments of additional osteoderms. Although this specimen includes fewer osteoderms than the two referred specimens, UMNH VP 28350 and UMNH VP 28351 (Fig. 2), it was selected as the holotype because its osteoderms are the best-preserved among the three specimens and most clearly exhibit two of the three features in the unique combination of characters that distinguishes *I. zephyri*.

##### Cervical/pectoral osteoderms

**Figure 3:**
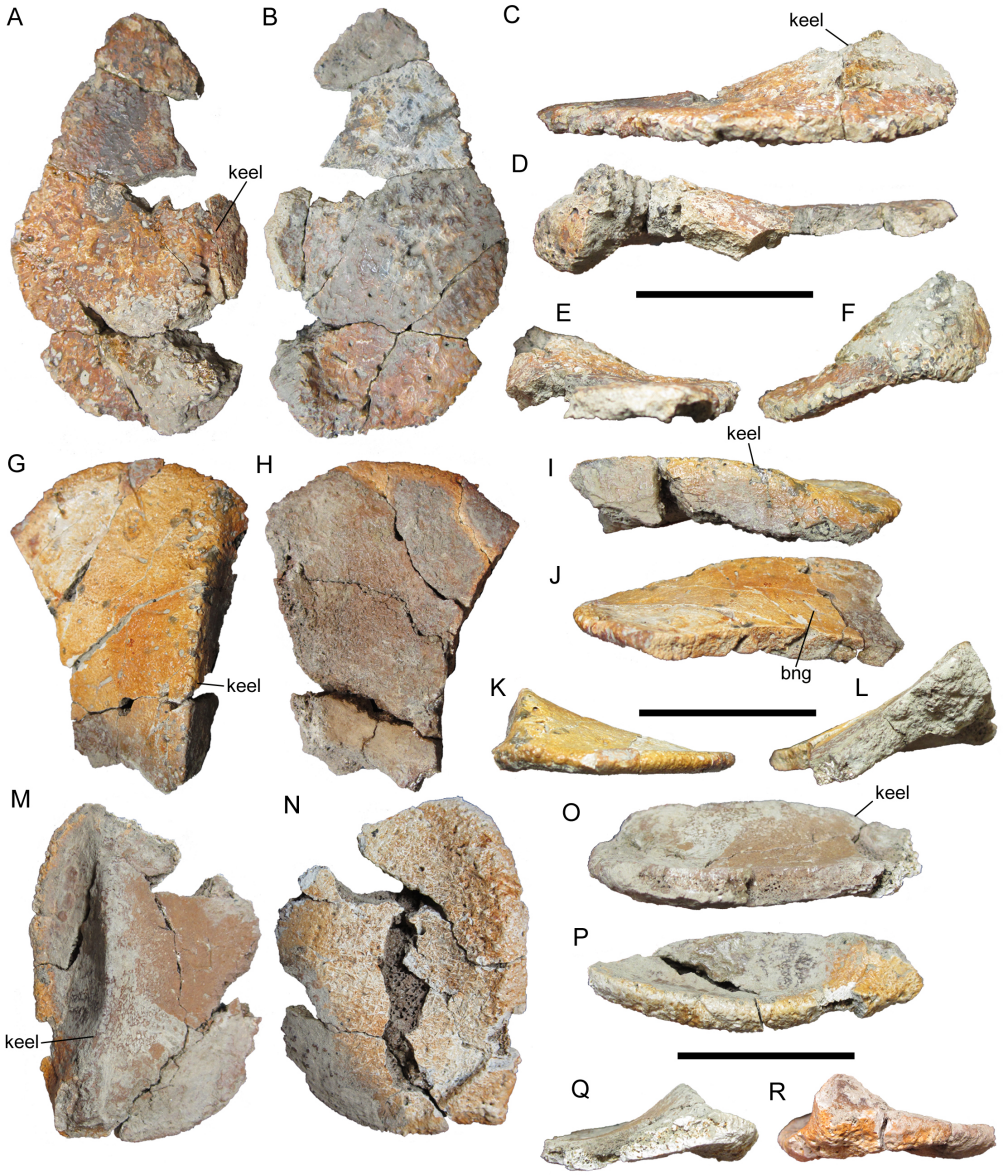
Cervical/pectoral osteoderms of WSC 16505, holotype of *I. zephyri*. WSC 16505.1, right medial cervical/pectoral osteoderm in (A) external, (B) basal, (C) medial, (D) lateral, (E) cranial, and (F) caudal views. WSC 16505.2, left medial cervical/pectoral osteoderm in (G) external, (H) basal, (I) medial, (J) lateral, (K) cranial, and (L) caudal views. WSC 16505.3, left distal osteoderm from the second cervical half-ring in (M) external, (N) basal, (O) medial, (P) lateral, (Q) cranial, and (R) caudal views. Study site: bng, bifurcated neurovascular groove. Cranial is toward the top of the figure in A, B, G, H, M, and N. Scale bars equal five cm.

Western Science Center 16505.1 is identified as a possible right medial cranial/pectoral osteoderm. The medial cervical/pectoral osteoderms of *Edmontonia rugosidens* (AMNH 5665) (Plates 5, 8 in Gilmore (1930)), *Panoplosaurus mirus* (Plates 5, 6 in Lambe (1919)), and *G. mimus* (Figs. 2 and 5B in Burns (2008)) have straight medial margins, suggesting that the straight preserved margin on WSC 16505.1 is the medial margin (Figs. 3A–3D). WSC 16505.1 preserves the caudal and medial margins, but is broken along the cranial and lateral margins (Figs. 3A–3F). The caudal margin is straight immediately medial to the caudal end of the keel, but then curves craniomedially into the straight medial margin, suggesting a rectangular or subrectangular shape reminiscent of the medial cervical/pectoral osteoderms of *Edmontonia rugosidens* (AMNH 5665) (Gilmore, 1930) and *G. mimus* (Morphotype F of Burns (2008)). Lateral to the keel, the caudal margin curves craniolaterally. The caudal margin is overall convex, with the caudal-most point on the osteoderm being the caudal end of the keel, as on the medial cervical/pectoral osteoderms of *E. rugosidens*, *P. mirus*, and *G. mimus*. The preserved margins are rugose, with an intricate morphology of furrows, small projecting bumps, and, at the caudal end of the keel, abundant neurovascular pits (Figs. 3A–3F). The osteoderm thins considerably toward its preserved margins. At the thickest preserved point on the keel, the osteoderm is 1.8 cm thick, while it is only 0.6 cm thick along its medial margin. This thinning occurs gradually, such that the medial flank of the keel exhibits a very gentle gradient and the keel is poorly defined compared to the rest of the osteoderm’s external surface. The keel itself is low, rounded, and thins cranially so that it appears not to have reached the cranial margin (Figs. 3A–3F).

Western Science Center 16505.1 is the only osteoderm in WSC 16505 that exhibits any degree of projecting rugosity on its external surface. The entire external surface is covered in small subcircular neurovascular pits of more or less uniform size, though a few larger pits occur adjacent to the caudal margin and on the caudal end of the keel (Figs. 3A and 3F). On the keel and near the caudal margin of the osteoderm, the pits are interspersed with small patches of projecting rugosity. This rugosity diminishes toward the cranial margin. Toward the cranial margin, the pitted texture continues, but the surfaces among the pits are smooth, as is the case on the entire external surfaces of all other osteoderms in WSC 16505. The transition from pitted and rugose to pitted and smooth texture is abrupt. There are no neurovascular grooves present on the preserved portion of the osteoderm.

The basal surface of this medial cervical/pectoral osteoderm is similar to the external in exhibiting a large number of small pits of uniform size distributed randomly over the entire surface (Fig. 3B). Projecting bumps are present on the basal surface as well, though compared to the external surface these bumps are more prominent, fewer in number, more widely spaced, and extend the entire preserved craniocaudal length of the osteoderm. The basal surface has a woven appearance, with visible structural fibers. Adjacent to the medial margin of the osteoderm, the basal surface is flat. However, lateral to this flat expanse, the basal surface becomes arched, with a broad, shallow concavity extending craniocaudally and corresponding to the keel on the external surface (Fig. 3B).

Western Science Center 16505.2 is identified as a medial cervical/pectoral osteoderm based upon similarities with the medial cervical/pectoral osteoderms of *Glyptodontopelta* (Fig. 5B in Burns (2008)), including a straight, sharp keel that is dorsally convex toward its cranial end (Figs. 3G–3L). The preserved portion of the cranial margin of WSC 16505.2 is gently convex, similar to the craniolateral margins of the left medial cervical/pectoral osteoderms of *Glyptodontopelta* (Figs. 2 and 5B in Burns (2008)) and different from the square craniomedial margins of those same osteoderms. This suggests that WSC 16505.2 is a left medial cervical/pectoral osteoderm. WSC 16505.2 is broken medially and caudally, and is missing most of its lateral margin (Figs. 3G–3L). The texture of the cranial margin is similar to that of the medial and caudal margins of WSC 16505.1, though with less pronounced rugosity. As in WSC 16505.1, WSC16505.2 becomes much thinner along its margins and away from its keel. At its greatest preserved depth, the keel’s apicobasal thickness is 2.2 cm. At the cranial margin, the osteoderm thins to only 0.6 cm. However, in contrast to WSC 16505.1, the keel of WSC 16505.2 reaches the cranial margin and is well demarcated along its entire preserved length except at the cranial margin, where it diminishes and merges with the margin (Figs. 3G and 3I–3K). The keel is more sharply defined on WSC 16505.2 than on WSC 16505.1, with steeper lateral and medial flanks.

The entire preserved external surface of WSC 16505.2 is akin to that of the cranial portion of WSC 16505.1: smooth with no rugosity and numerous, randomly distributed, small pits (Fig. 3G). Most of the pits on WSC 16505.2 are miniscule, smaller than those on WSC 16505.1, although larger pits are present along the apex of the keel. WSC 16505.2 also differs from WSC 16505.1 in the presence of a small number of randomly distributed neurovascular grooves. Some of these grooves are simple, non-branching furrows, while others bifurcate, forming a Y-shaped groove with the opening of the “Y” directed laterally (Fig. 3G).

The basal surface of WSC 16505.2 lacks the projecting bumps present on WSC 16505.1. Otherwise, the basal surfaces of the two osteoderms are similar, with abundant small pits and a broad, shallow groove extending craniocaudally and corresponding to the keel on the external surface (Fig. 3H).

Western Science Center 16505.3 is identified as the distal osteoderm of the left side of the second cervical half-ring. It is nearly complete, missing only some small portions of the medial margin (Figs. 3M–3R). This osteoderm is more oval in shape than WSC 16505.1 and 16505.2, making it difficult to delineate the cranial and caudal margins from the medial margin. In contrast, the lateral margin is easily demarcated; the cranial-most margin of the osteoderm is incomplete, but the caudal-most margin is preserved and forms an abrupt, only slightly obtuse angle with the lateral margin. The preserved margins are similar to the rugose margins of WSC 16505.1 and 16505.2.

The keel of this distal cervical osteoderm is strongly laterally offset, as on the distal osteoderms of the second cervical half-ring of *E. rugosidens* (AMNH 5665). However, the distal cervical osteoderm of *I. zephyri* differs from those of AMNH 5665 in that the keel curves laterally, rather than medially, toward its cranial end (Fig. 3M). The keel is very pronounced, with a steep medial flank and precipitous, nearly vertical lateral flank. At its deepest point, the keel is 2.2 cm thick. At a point directly lateral to this along the lateral margin, the osteoderm is only 0.8 cm thick.

The external surface of this distal cervical osteoderm is very similar to that of WSC 16505.2: smooth with numerous miniscule pits and a smaller number of larger pits along the apex of the keel, and a small number of randomly distributed neurovascular grooves (Fig. 3M). The basal surface also is similar in texture to that of WSC 16505.2, with abundant small pits randomly distributed over the entire surface and a broad, shallow, longitudinal concavity corresponding to the position of the keel on the external surface. WSC 16505.3 measures 6.3 cm at its greatest mediolateral width and 8.4 cm at its greatest craniocaudal length.

##### Thoracic osteoderms

The life placements of ankylosaur thoracic osteoderms can be inferred from the position of the keel, as noted by Burns (2008). A keel located at or near the midline of an osteoderm indicates that the osteoderm was positioned near the animal’s midline. A keel offset to the right or left, resulting in an asymmetrical osteoderm, indicates that the osteoderm was positioned more laterally. Three distinct morphotypes are present among the thoracic osteoderms of WSC 16505.

**Figure 4:**
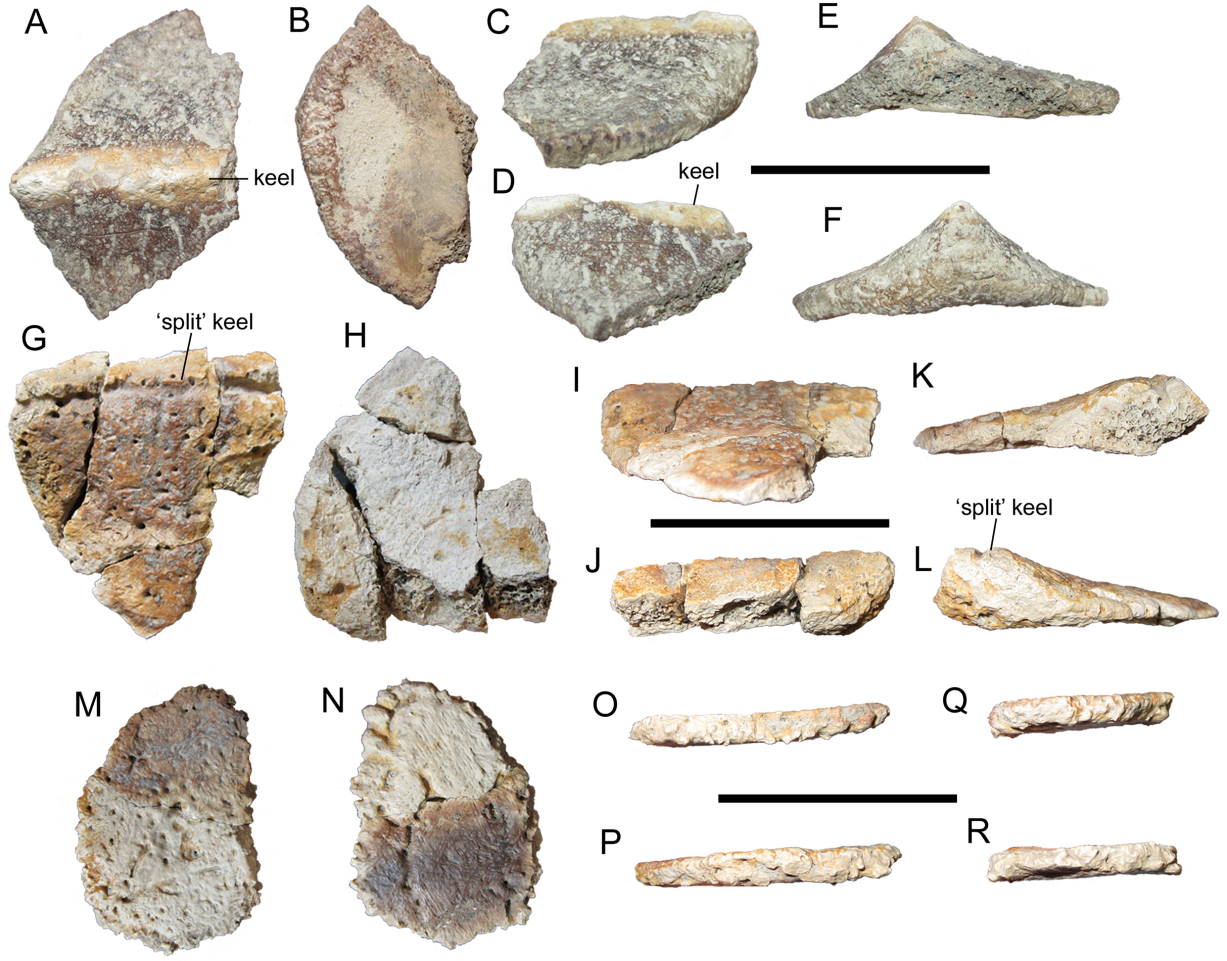
Thoracic osteoderms of WSC 16505, holotype of *I. zephyri*. WSC 16505.4, lateral thoracic osteoderm in (A) external, (B) basal, (C) medial, (D) lateral, (E) cranial, and (F) caudal views. WSC 16505.5, thoracic osteoderm in (G) external, (H) basal, (I) medial or lateral (orientation uncertain), (J) medial or lateral (orientation uncertain), (K) cranial, and (L) caudal views. WSC 16505.6, thoracic ossicle in (M) external, (N) basal, and (O–R) marginal views (orientation uncertain). Scale bars equal five cm.

Thoracic osteoderm WSC 16505.4 is incomplete cranially but appears similar to Morphotype A of Burns (2008), though perhaps with a more prominent keel (Figs. 4A–4F). The caudal margin of this osteoderm is moderately rugose, similar to the margins of WSC 16505.2 and 16505.3 (Figs. 3G–3R). At the caudal end of the keel, situated at the caudal margin of the osteoderm, the osteoderm is 1.8 cm thick. The offset of the keel indicates that this osteoderm was situated laterally.

The external surface texture of WSC 16505.4 is consistent with the cervical/pectoral osteoderms. The texture is smooth, with numerous neurovascular pits of roughly the same size randomly distributed from the keel to the margins. Three subparallel non-branching neurovascular grooves are present lateral to the keel (Fig. 4A); a single non-branching groove is discernible medial to the keel. This is reminiscent of the characteristic dendritic pattern of neurovascular grooves on the osteoderms of *G. mimus* (Burns, 2008); however, none of the osteoderms of WSC 16505 exhibit the density of radiating neurovascular grooves to both sides of the keel exhibited by *G. mimus* (Figs. 4–6 in Burns (2008)). The basal surface is not as well preserved as the external, though it was clearly gently concave (Fig. 4B).

Another large thoracic osteoderm, WSC 16505.5, was apparently similar in shape to WSC 16505.4, at least in having a rounded caudal margin (Figs. 4G–4L). The keel of WSC 16505.5 is low, rounded, and poorly defined, unlike that of WSC 16505.4. The subtlety of the keel of WSC 16505.5 is reminiscent of Morphotype B in *G. mimus* (Burns, 2008). However, the keel of WSC 16505.5 differs from those of *Glyptodontopelta* and other nodosaurids in having a “split” morphology, with a deep, sharply defined groove extending craniocaudally along the apex of the keel (Fig. 4G). The smooth, copiously pitted external surface texture continues uninterrupted into this groove, with no deformation that might signify a pathological nature. This same feature is also present on a thoracic osteoderm of UMNH VP 28351, one of the specimens referred to *Invictarx* (see Thoracic osteoderms of UMNH VP 28351 below). This feature is also present on some thoracic osteoderms of the ankylosaurids *A. lambei* and *P. coombsi* (Figs. 13G and 13O in Penkalski (2018)) (P. Penkalski, 2018, personal communication).

The external surface texture of WSC 16505.5 closely resembles that of the other osteoderms in the specimen, with a smooth surface invaded by numerous, randomly distributed pits of uniform size (Fig. 4G). A single short neurovascular groove branches off from the elongate groove that forms the “split-keel” morphology. The basal surface is nearly featureless, except for a small number of randomly distributed pits (Fig. 4H). The basal surface is flat apart from a very slight arching ventral to the keel.

The only complete thoracic osteoderm, WSC 16505.6, is also the most puzzling. It is quite small compared to the other osteoderms in WSC 16505, and fits the definition of an “ossicle” as proposed by Burns & Currie (2014): “small (<70 mm), amorphous mineralized dermal elements often found interstitial to major osteodermal elements.” WSC 16505.6 measures only 5.1 cm across at its maximum width. This ossicle is uniformly very thin, only 0.5 cm thick at its center. The external surface is very slightly concave with no trace of a keel, while the basal surface is flat (Figs. 4M–4R). WSC 16505.6 is quite similar in size and shape to Morphotype D in *G. mimus* (Burns, 2008). Despite their thinness, the margins of WSC 16505.6 are highly rugose, similar to those of WSC 16505.1, the medial cervical/pectoral osteoderm described above (Figs. 3A–3F). The external and basal surface textures of WSC 16505.6 are like those of the other osteoderms in WSC 16505, especially the larger thoracic osteoderm WSC 16505.5 (Figs. 4M and 4N). There are two well-defined neurovascular grooves on the external surface, one non-branching and the other bifurcating.

##### Comparative osteoderm surface texture

As described above, there is some variation in surface texture among the osteoderms of WSC 16505. However, each individual cervical/pectoral or thoracic osteoderm conforms to the overall pattern of having a smooth surface with little or no projecting rugosity, abundant pits distributed randomly over the entire external surface, and either no neurovascular grooves or a small number of bifurcating and non-bifurcating neurovascular grooves distributed randomly.

The osteoderms of WSC 16505 are similar in gross morphology to those of other nodosaurids. However, details of the external surface textures are different. Although the cervical/pectoral and thoracic osteoderms of *E. rugosidens* (AMNH 5665, TMP 1998.98.1, National Museum of Natural History (USNM) 11868; Gilmore, 1930; Burns, 2008; Burns & Currie, 2014), *P. mirus* (Lambe, 1919; Burns, 2008; Burns & Currie, 2014), and *S. landerensis* (Field Museum of Natural History (FMNH) UR88; Moodie, 1910) exhibit a heavily pitted (“scrobiculate”; Moodie, 1910) texture, they all have some degree of projecting rugosity, in contrast to the pitted but smooth texture of WSC 16505. The osteoderms of *G. mimus* display abundant pitting with smooth surfaces among the pits (Burns, 2008), similar to WSC 16505. However, WSC 16505 lacks the dense pattern of dendritic grooves that characterizes the osteoderms of *Glyptodontopelta* (Burns, 2008; Burns & Currie, 2014). Furthermore, none of the known osteoderms of *Glyptodontopelta* exhibit the “split-keel” morphology of WSC 16505.5.

#### Description of UMNH VP 28350

Natural History Museum of Utah VP 28350 is an incomplete, associated postcranial skeleton that includes three dorsal vertebrae, many small fragments of dorsal ribs, distal end of the left humerus, distal end of the left ulna, proximal ends of the left and right radii, incomplete metacarpal, several incomplete but identifiable osteoderms, and fragments of additional osteoderms. Measurements of select axial and appendicular elements of UMNH VP 28350 are available in the supplementary information (Table S1 of Measurements).

##### Dorsal vertebrae

Natural History Museum of Utah VP 28350 includes three incomplete but well-preserved dorsal vertebrae. One consists of only the centrum, but the other two preserve the base of the neural arch and partial prezygapophyses. Based upon comparisons with nodosaurids that have more complete and fully described dorsal series, especially *S. edwardsorum* (Ostrom, 1970) and *E. carbonensis* (Kirkland et al., 2013), the three vertebrae of UMNH VP 28350 are identified as middle dorsals. There are no indications of dorsal ribs fused to these vertebrae.

**Figure 5:**
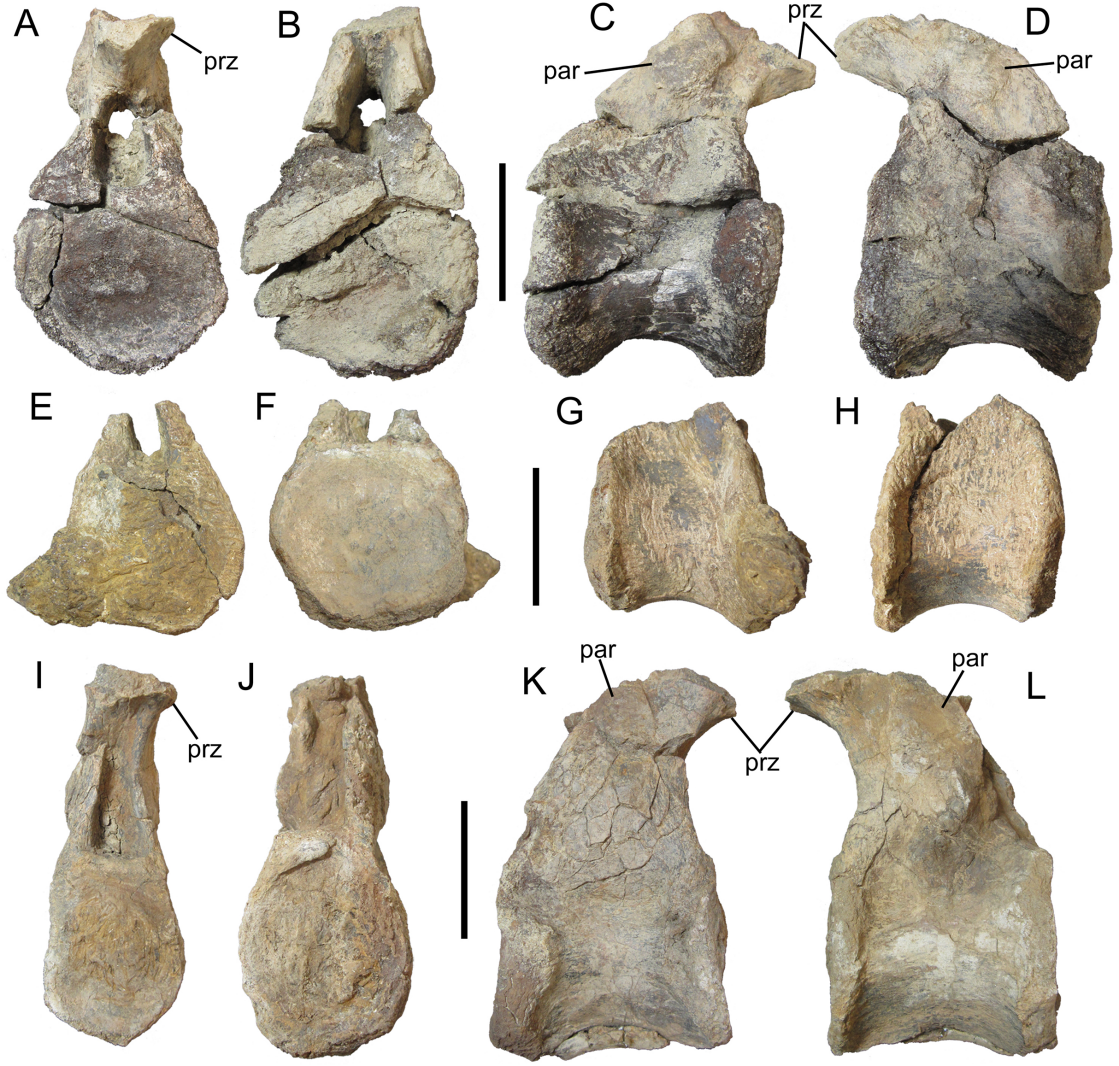
Dorsal vertebrae of UMNH VP 28350, referred specimen of *I. zephyri*. Middle dorsal vertebra in (A) cranial, (B) caudal, (C) right lateral, and (D) left lateral views. Middle dorsal vertebra in (E) cranial, (F) caudal, (G) right lateral, and (H) left lateral views. Middle dorsal vertebra in (I) cranial, (J) caudal, (K) right lateral, and (L) left lateral views. Study sites: par, parapophysis; prz, prezygapophysis. Scale bars equal five cm.

The vertebrae exhibit amphiplatyan centra, with the cranial and caudal faces only slightly concave (Figs. 5A, 5B, 5E, 5F, 5I and 5J), as in *Sauropelta* (Ostrom, 1970) and *Europelta* (Kirkland et al., 2013). The cranial and caudal faces are subcircular. In lateral view, the neural arch rises vertically from the craniodorsal margin of the centrum, forming a nearly right angle with the long axis of the centrum (Figs. 5C, 5D, 5G, 5H, 5K and 5L); the caudal margin of the neural arch forms a much gentler slope relative to the long axis of the centrum. The parapophyses are distinct, rugose swellings on the lateral surfaces of the neural arch (Figs. 5C, 5D, 5K and 5L). The prezygapophyses are joined ventrally and form a nearly horizontal, craniocaudally short parapet on the cranial margin of the neural arch above the neural canal. Due to breakage, the shape of the articular facets cannot be determined. The neural canal itself is elliptical in cranial and caudal views, with its long axis oriented dorsoventrally (Figs. 5A, 5B, 5I and 5J), as in *Sauropelta* (Ostrom, 1970), *Europelta* (Kirkland et al., 2013), *Silvisaurus* (Carpenter & Kirkland, 1998), and *S. austriacus* (Pereda Suberbiola & Galton, 2001). It is somewhat broader along its ventral margin than along its dorsal. The diapophyses and neural spines are not preserved.

##### Humerus

**Figure 6:**
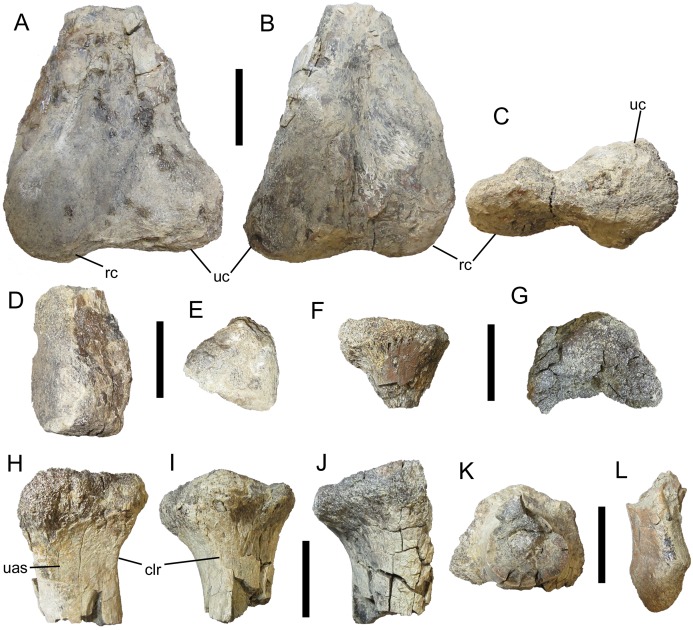
Appendicular elements of UMNH VP 28350, referred specimen of *I. zephyri*. Distal end of left humerus in (A) cranial, (B) caudal, and (C) distal views. Distal end of left ulna in (D) medial and (E) distal views. (F) Proximal end of left radius, orientation uncertain. Proximal end of right radius in (G) proximal, (H) lateral, (I) cranial, (J) medial, and (K) distal views. (L) Metacarpal, orientation uncertain. Study sites: clr, craniolateral ridge; rc, radial condyle; uas, articulation surface for ulna; uc, ulnar condyle. Scale bars equal five cm.

The appendicular elements of UMNH VP 28350 are all incomplete and poorly preserved; however, comparison with other nodosaurids, particularly the articulated forelimb of *Niobrarasaurus coleii* (Carpenter, Dilkes & Weishampel, 1995), has facilitated tentative identifications. The distal end of the left humerus is crushed and lacking much of the bone surface. Nevertheless, it is clear that the ulnar condyle was larger than the radial condyle, occupying more of the cranial and caudal surfaces of the distal end of the humerus (Figs. 6A and 6B). Although incomplete, the radial condyle appears to have had the subspherical shape characteristic of nodosaurids (Coombs, 1978; Vickaryous, Maryańska & Weishampel, 2004), and would match the probable circular shape of the proximal end of the radius (see below). The ulnar and radial condyles are separated by a smooth, shallow cleft (Fig. 6C).

##### Ulna

The distal end of the left ulna also is in poor condition, broken and stripped of most of its surface. In overall shape, it resembles the distal ends of the ulnae of *S. edwardsorum* (Plate 24E, F in Ostrom (1970)) and *Niobrarasaurus coleii* (Figs. 10B and 10C in Carpenter, Dilkes & Weishampel (1995)) (Fig. 6D). The distal articulation surface is mediolaterally expanded and rugose, with a pattern of short, non-branching, parallel grooves discernible along its craniomedial margin (Fig. 6E).

##### Radii

The partial proximal ends of both radii are present, with the right radius more complete than the left, which consists of only a fragment that reveals little about the element’s morphology (Fig. 6F). In contrast, the proximal end of the right radius, though broken caudally, is the best-preserved of the appendicular elements of UMNH VP 28350 (Figs. 6G–6K). The expanded proximal articulation surface is shallowly concave and was probably circular (Fig. 6G), as is typical of nodosaurids (Coombs, 1978; Vickaryous, Maryańska & Weishampel, 2004). Distal to the proximal articulation surface, the lateral surface of the shaft of the radius forms a nearly flat surface for articulation with the ulna; this surface is demarcated cranially by a subtle ridge that extends proximodistally along the craniolateral surface of the shaft (Figs. 6H and 6I). The shaft itself is subcircular in cross-section (Fig. 6K).

##### Metacarpal

Natural History Museum of Utah VP 28350 includes an incomplete metacarpal lacking the proximal and distal ends (Fig. 6L). Little can be determined regarding the morphology, orientation, and placement of this fragment.

##### Cervical/pectoral osteoderms

**Figure 7:**
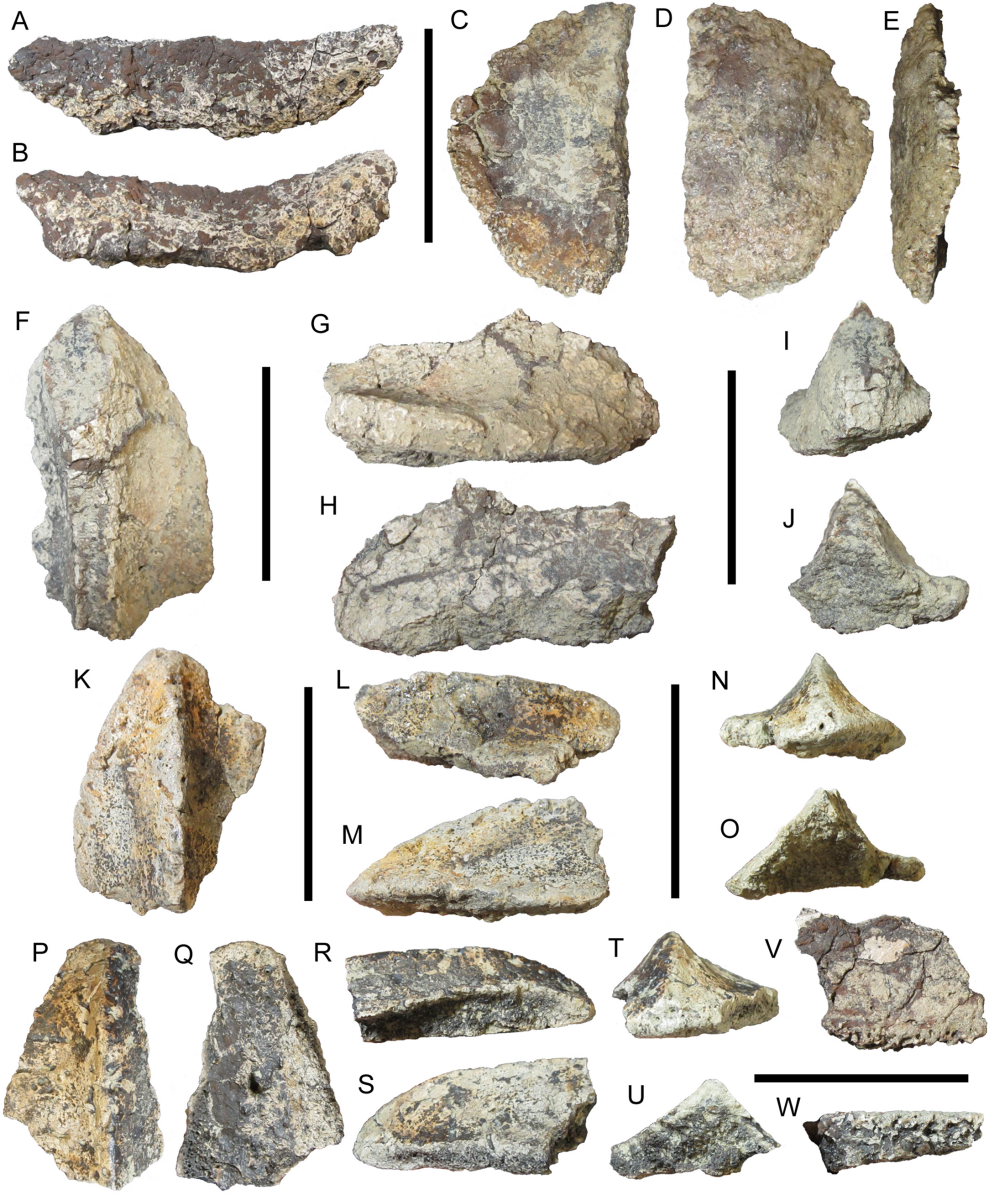
Osteoderms of UMNH VP 28350, referred specimen of *I. zephyri*. Cervical/pectoral osteoderm fragment in (A) external view and (B) medial or lateral view (orientation uncertain). Distal osteoderm of second cervical half-ring in (C) external, (D) basal, and (E) medial views. Dorsal thoracic osteoderm in (F) external view, (G) medial or lateral view (orientation uncertain), (H) medial or lateral view (orientation uncertain), (I) cranial or caudal view (orientation uncertain), and (J) cranial or caudal view (orientation uncertain). Lateral thoracic osteoderm in (K) external, (L) medial, (M) lateral, (N) cranial, and (O) caudal views. Lateral thoracic osteoderm in (P) external, (Q) basal, (R) lateral, (S) medial, (T) cranial, and (U) caudal views. Pelvic osteoderm fragment in (V) external view and (W) marginal view (orientation uncertain). Scale bars equal five cm.

Natural History Museum of Utah VP 28350 includes two fragments identifiable as partial components of the cervical/pectoral half-rings. One of these is a large sliver that probably represents the medial or lateral margin of a broad, rounded plate (Fig. 7A). The preserved margin is highly rugose with numerous pits and projecting rugosity (Fig. 7B).

The other cervical/pectoral osteoderm fragment of UMNH VP 28350 is a simple semicircular piece (Figs. 7C and 7D). The external surface is shallowly concave, while the basal surface is gently convex. Although the external surface is partially coated with siderite, the visible areas of texture are consistent with the cervical/pectoral osteoderms of WSC 16505 in exhibiting smooth texture with little or no projecting rugosity and numerous pits of random distribution, supporting referral of UMNH VP 28350 to *I. zephyri*. The preserved margins are highly rugose (Fig. 7E). An almost straight break extends the length of this fragment on one side. No keel is present on the preserved portion of this osteoderm. The shape of this osteoderm resembles the portion of WSC 16505.3 medial to the keel (Figs. 3M, 3N and 3P), suggesting that this osteoderm is also a distal osteoderm of the second cervical half-ring.

##### Thoracic osteoderms

The largest thoracic osteoderm of UMNH VP 28350 is incomplete, but preserves part of a very prominent broken keel and part of either the medial or lateral margin (Figs. 7F–7H). The external surface is not well preserved, but appears to have been similar to those of the osteoderms of WSC 16505—smooth with numerous small pits of random distribution and no projecting rugosity. The basal surface is not preserved at all. The apparent symmetry of the available portion of this osteoderm indicates it probably was located near the animal’s midline (Figs. 7I and 7J). The preserved portion of this osteoderm suggests it might have been a craniocaudally elongate element similar to some of the osteoderms of *E. carbonensis* (Types D and E of Kirkland et al. (2013)).

Natural History Museum of Utah VP 28350 includes two partial but well-preserved asymmetrical thoracic osteoderms with offset keels, indicating that they were positioned more laterally in life (Figs. 7K–7U). Where preserved, the margins of these lateral thoracic osteoderms are smoother than those of the cervical/pectoral osteoderms. The keels are very pronounced, giving these osteoderms cross-sections shaped like scalene triangles (Figs. 7O and 7U). The external surfaces of these osteoderms closely resemble those of the cervical/pectoral and thoracic osteoderms of WSC 16505. The external surface is quite smooth, lacking any projecting rugosity. Small neurovascular pits of uniform size are randomly distributed across the surface, from the apex of the keel to the margins. A small number of non-bifurcating neurovascular grooves are randomly distributed to one side of the keel (Figs. 7K and 7P). The well-preserved external surface texture on these two lateral thoracic osteoderms links UMNH VP 28350 to WSC 16505 and supports referral to *I. zephyri*. The basal surface texture is preserved on only one of these osteoderms. It is nearly flat and exhibits numerous small pits. One much larger neurovascular foramen is present, extending obliquely into the osteoderm (Fig. 7Q).

##### Pelvic osteoderms

Two fragments are identified as pieces of pelvic osteoderms. Both fragments have flat external and basal surfaces with small, randomly distributed pits (Fig. 7V). Each fragment preserves part of a margin. Unlike the cervical/pectoral and thoracic osteoderms of WSC 16505 and UMNH VP 28350, these fragments do not become thinner toward the margins; instead, the thicknesses of the osteoderms remain constant (Fig. 7W). The preserved margins on the two pelvic osteoderm fragments are extremely thick compared to the margins of the cervical/pectoral and thoracic osteoderms; the greatest thickness of the margin on the larger of the two fragments is 1.2 cm. In contrast, the greatest marginal thickness of one of the lateral thoracic osteoderms described above is 0.6 cm (Figs. 7K–7M). The available morphology of these pelvic osteoderm fragments (flat external and basal surfaces, and thick, non-tapering margins) matches the Morphotype C osteoderms of *G. mimus* (Burns, 2008). In *Glyptodontopelta*, osteoderms of this type comprise a co-ossified pelvic shield consisting of polygonal osteoderms of uniform size (Category 3 pelvic shield of Arbour, Burns & Currie (2011)). UMNH VP 28351, the other referred specimen of *I. zephyri*, provides additional and stronger evidence for the presence of a Category 3 co-ossified pelvic shield (see Pelvic osteoderms of UMNH VP 28351 below).

#### Description of UMNH VP 28351

Natural History Museum of Utah VP 28351 includes several fragmentary vertebral centra, fragments of dorsal ribs, several identifiable osteoderms, and numerous additional osteoderm fragments. The severely weathered and broken centra provide no morphological information to supplement the descriptions of the better preserved vertebrae of UMNH VP 28350 (see above). Although UMNH VP 28351 includes the largest number of osteoderms of the three specimens of *I. zephyri*, all are incomplete and many are coated in a veneer of siderite or have been stripped of their external and basal surface textures. Nevertheless, UMNH VP 28351 provides valuable information not available in WSC 16505 or UMNH VP 28350, particularly the presence of pectoral/thoracic spines and the morphology of the pelvic osteoderms.

##### Cervical/pectoral osteoderms

**Figure 8:**
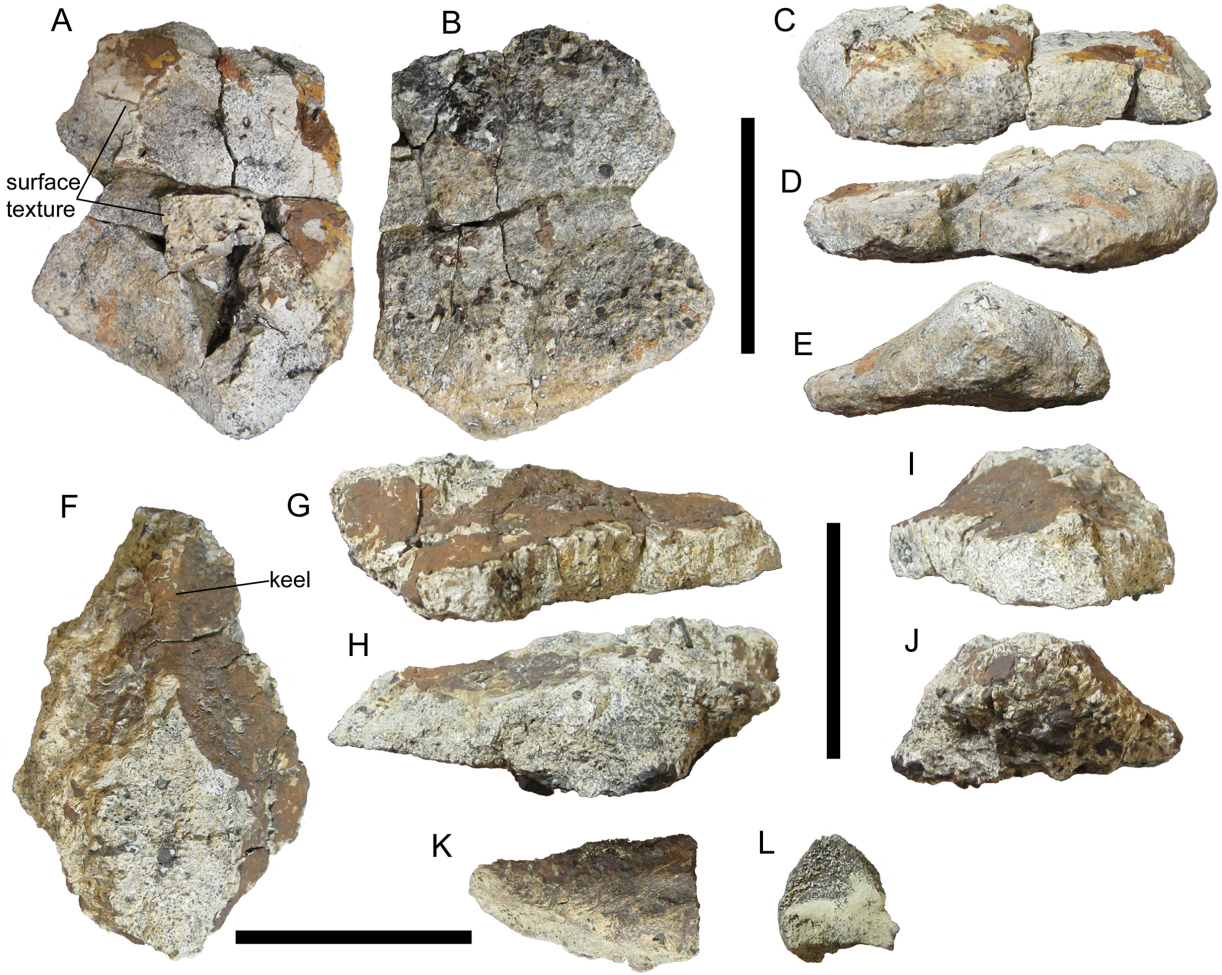
Cervical/pectoral osteoderms of UMNH VP 28351, referred specimen of *I. zephyri*. Medial cervical/pectoral osteoderm in (A) external, (B) basal, (C) medial or lateral (orientation uncertain), (D) medial or lateral (orientation uncertain), and (E) caudal views. Base of pectoral or thoracic distal spine in (F) external, (G) medial or lateral (orientation uncertain), (H) medial or lateral (orientation uncertain), (I) cranial, and (J) caudal views. Tip of pectoral or thoracic distal spine in (K) view of preserved external surface and (L) view of cross-section. Cranial is toward the top of the Figure in A, B, and F. Scale bars equal five cm.

Natural History Museum of Utah VP 28351 includes a broad, thick plate that most likely pertains to one of the cervical/pectoral half-rings. This osteoderm is broken on all sides apart from the inferred caudal margin. The caudal margin is not straight, but rather comes to a rounded protrusion (Figs. 8A and 8B). This morphology is also present on the medial cervical/pectoral osteoderms of *P. mirus* (Lambe, 1919), *E. rugosidens* (AMNH 5665) (Gilmore, 1930), and *G. mimus* (Burns, 2008). Furthermore, in its indistinct, gentle keel and cross-sectional shape this osteoderm of UMNH VP 28351 closely matches WSC 16505.1, a right medial cervical/pectoral osteoderm, albeit much thicker (Figs. 3F and 8C–8E). Unlike many of the osteoderms of UMNH VP 28351, this medial cervical/pectoral osteoderm preserves patches of the external surface texture. This closely resembles that of WSC 16505 in being smooth with numerous neurovascular pits (Fig. 8A), supporting referral of UMNH VP 28351 to *I. zephyri*. Short, non-branching grooves are also present on these patches of surface texture. The basal surface texture is not well preserved, but sideritic infilling has revealed the presence of abundant pitting (Fig. 8B).

Natural History Museum of Utah VP 28351 includes another very thick osteoderm that is unlike any of the osteoderms of WSC 16505 and UMNH VP 28350. This osteoderm is broken on all sides. A siderite coating has obscured the external surface texture, and the basal surface texture has been weathered away (Fig. 8F). Nevertheless, the discernible morphology of this osteoderm allows some precision as to its identification. A low but distinct keel is present on the external surface. Caudally, this keel becomes much wider mediolaterally and the osteoderm overall becomes much thicker, forming an oval pedestal that is truncated by a break (Figs. 8G–8J). This morphology resembles the bases of the cervical/pectoral and thoracic spines of *E. rugosidens* (AMNH 5665, TMP 1998.98.1, USNM 11868; Gilmore, 1930). *E. rugosidens* is unusual in having four osteoderms on either side of the second cervical half-ring—broad, plate-like medial, lateral, and distal osteoderms, and a craniolaterally-directed spine (AMNH 5665) (Gilmore, 1930). In other nodosaurids for which all three cervical/pectoral half-rings are preserved, there are only three osteoderms on either side of the second cervical half-ring; this is the case in *S. condrayi* (Eaton, 1960; Carpenter & Kirkland, 1998), *S. edwardsorum* (Carpenter & Kirkland, 1998), and *B. markmitchelli* (Brown et al., 2017). If WSC 16505.3 is correctly identified as the left distal osteoderm of a second cervical half-ring, then it is likely that the spine of UMNH VP 28351 was in the distal position on one side of the pectoral half-ring or the first thoracic band. *E. rugosidens* (AMNH 5665) (Gilmore, 1930) and *B. markmitchelli* (Brown et al., 2017) have distal spines on both the pectoral half-ring and first thoracic band.

Natural History Museum of Utah VP 28351 includes another fragment that probably belonged to a distal spine. This piece is a tapering prong with a D-shaped cross-section and inflated sides (Figs. 8K and 8L). External bone surface is present on only one of the three sides; this resembles the external surface texture of the other osteoderms of WSC 16505, UMNH VP 28350, and UMNH VP 28351 in having numerous small pits distributed over an otherwise smooth surface. Based upon comparisons with the spines of *E. rugosidens* (e.g., AMNH 5665, TMP 1998.98.1), this fragment is interpreted as the tip of a spine. It is impossible to ascertain whether it is part of the same spine as the osteoderm described above.

##### Thoracic osteoderms

**Figure 9:**
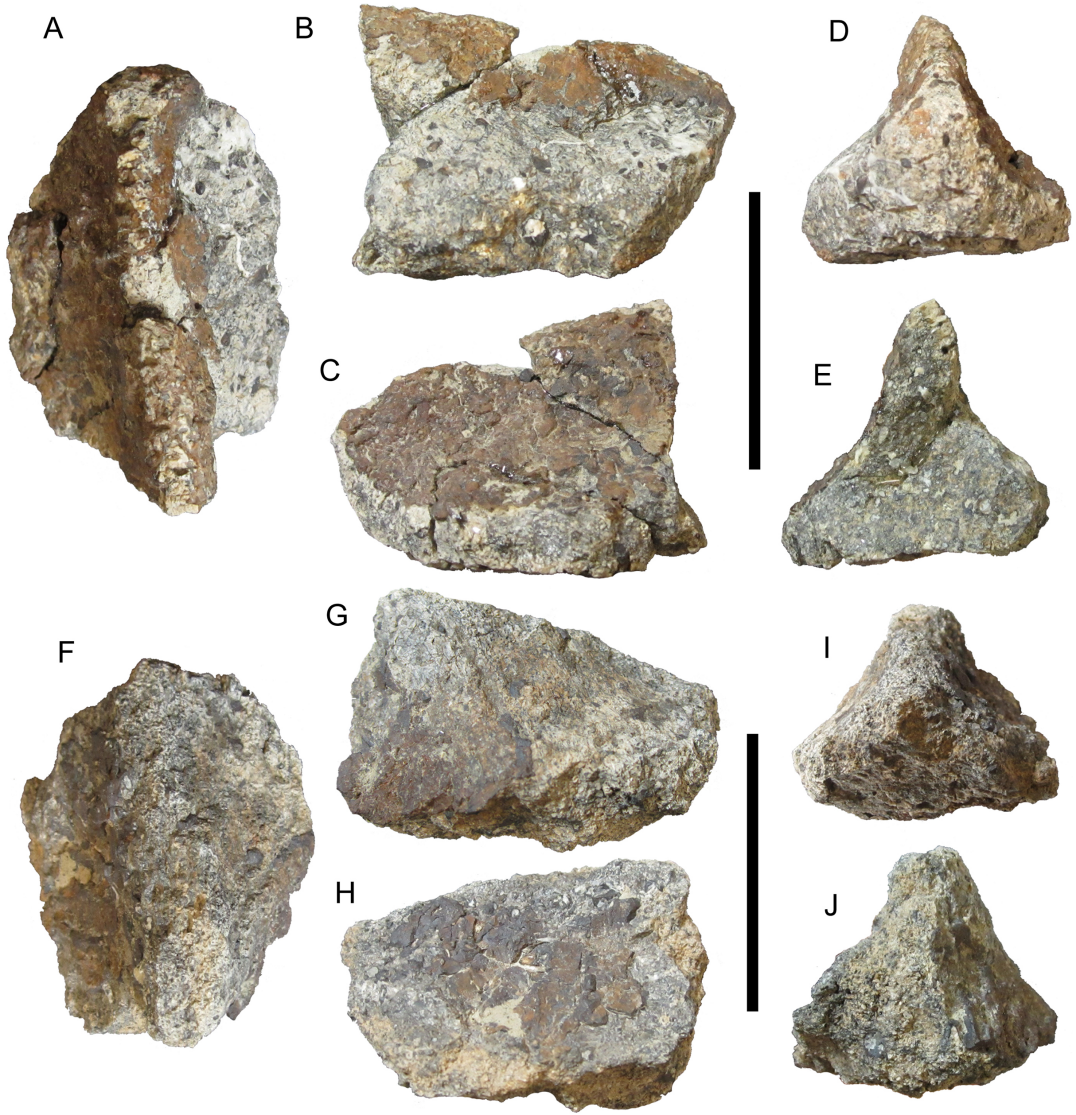
Thoracic osteoderms of UMNH VP 28351, referred specimen of *I. zephyri*. Dorsal thoracic osteoderm in (A) external, (B) medial or lateral (orientation uncertain), (C) medial or lateral (orientation uncertain), (D) cranial, and (E) caudal views. Dorsal thoracic osteoderm in (F) external, (G) medial or lateral (orientation uncertain), (H) medial or lateral (orientation uncertain), (I) cranial, and (J) caudal views. Scale bars equal five cm.

There are four morphotypes distinguishable among the thoracic osteoderms of UMNH VP 28351, all of which are also represented among the osteoderms of WSC 16505 and UMNH VP 28350. The first thoracic morphotype includes two partial osteoderms and at least two additional fragments. The two partial osteoderms are very thick with flat bases and sharp, prominent midline keels (Fig. 9). Due to weathering and siderite coating, details of the margins and external and basal surface textures cannot be discerned. However, in cross-sectional shape, these two osteoderms are similar to the large, craniocaudally elongate thoracic osteoderm of UMNH VP 28350, despite breakage of the apex of the keel on the osteoderm of UMNH VP 28350 (Figs. 7F–7J, 9D, 9E, 9I and 9J). Like the osteoderm of UMNH VP 28350, these two thoracic osteoderms of UMNH VP 28351 appear to have been craniocaudally elongate elements similar to Types D and E osteoderms of *E. carbonensis* (Kirkland et al., 2013). The midline positions of the keels of these two osteoderms indicate that they were probably situated near the midline of the animal. They are so similar in size and morphology that they might even constitute a left-right pair positioned parasagitally, as in the in situ thoracic bands of *Borealopelta markmitchelli* (Brown et al., 2017).

**Figure 10:**
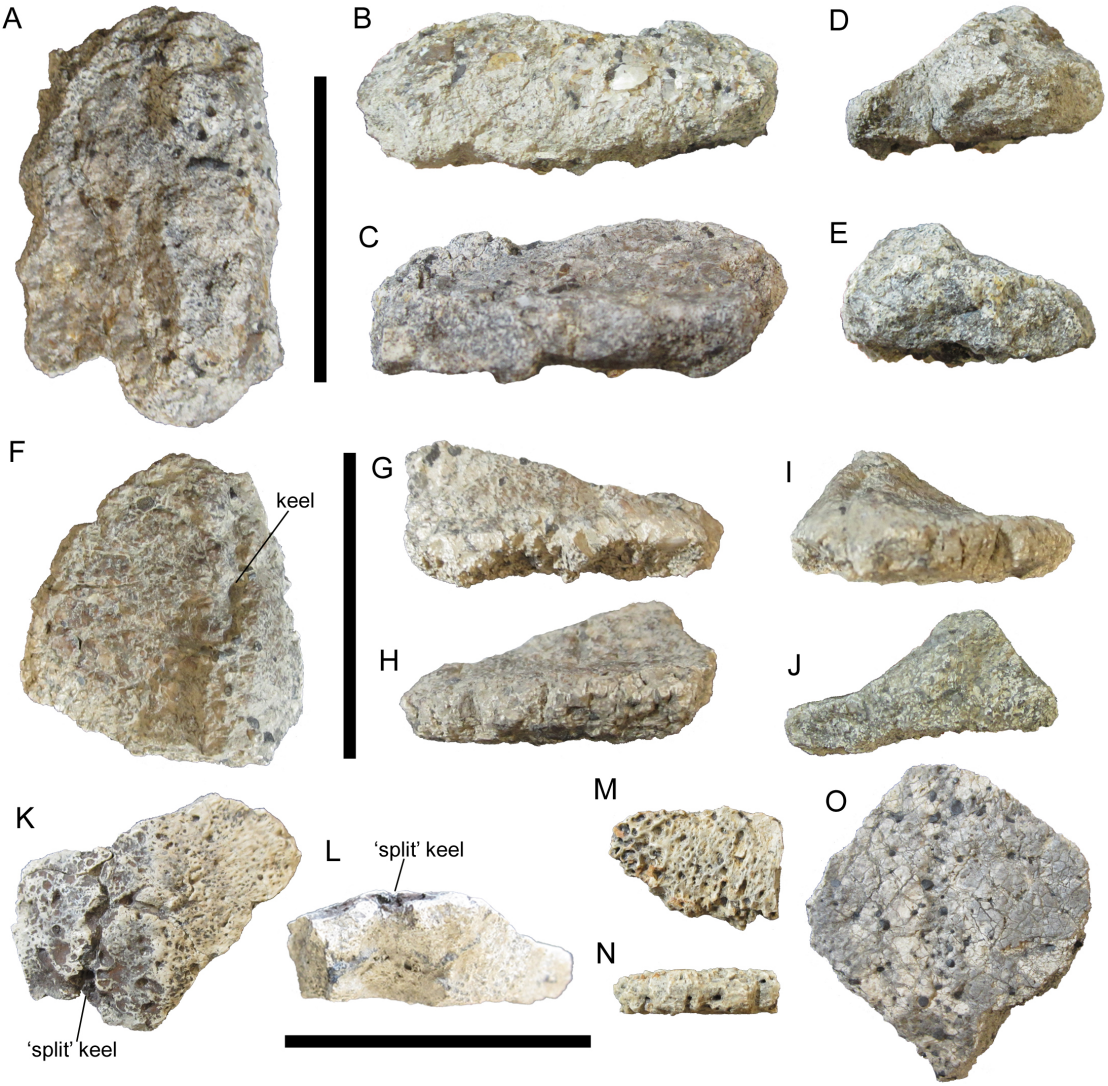
Thoracic osteoderms of UMNH VP 28351, referred specimen of *I. zephyri*. Lateral thoracic osteoderm in (A) external, (B) medial or lateral (orientation uncertain), (C) medial or lateral (orientation uncertain), (D) cranial or caudal (orientation uncertain), and (E) cranial or caudal (orientation uncertain) views. Lateral thoracic osteoderm in (F) external, (G) lateral, (H) medial, (I) cranial, and (J) caudal views. Thoracic osteoderm fragment in (K) external and (L) cross-sectional views. Thoracic interstitial ossicle in (M) external and (N) marginal views. Thoracic interstitial ossicle in (O) external view. Scale bars equal five cm.

The next thoracic morphotype observable in UMNH VP 28351 consists of more laterally positioned osteoderms with offset keels. This morphotype appears to be the most abundant class of osteoderm in UMNH VP 28351, with numerous fragments exhibiting small segments of offset keels. However, only two examples are sufficiently complete for meaningful comparison with the osteoderms of WSC 16505 and UMNH VP 28350. One example bears a strong resemblance in cross-sectional shape to the two well-preserved lateral thoracic osteoderms of UMNH VP 28350 (Figs. 7K–7U and 10A–10E). The apex of the keel and all the margins are broken in the osteoderm of UMNH VP 28351, and the external and basal surface textures are not preserved. The other example of this thoracic morphotype probably is the cranial end of an osteoderm very similar to WSC 16505.4, the caudal portion of a lateral thoracic osteoderm in the holotype of *I. zephyri* (see Thoracic osteoderms of WSC 16505 above). WSC 16505.4 and the osteoderm of UMNH VP 28351 are alike in cross-sectional shape and thickness, the prominence of the offset keel, and moderately rugose medial margin (Figs. 4A, 4C, 4E, 4F and 10F–10J). The keel of the UMNH VP 28351 osteoderm diminishes cranially, while the keel of WSC 16505.4 remains prominent up to the caudal margin.

The next thoracic morphotype is represented by only one definite example, which is an osteoderm fragment broken on all sides but exhibiting well-preserved external surface texture. It is similar to WSC 16505.5, a thoracic osteoderm of uncertain placement in the holotype of *I. zephyri*. WSC 16505.5 and the UMNH VP 28351 osteoderm share nearly flat bases; overall smooth external surface texture with numerous small pits of roughly uniform size and random distribution; and a low, rounded keel (Figs. 4G–4L, 10K and 10L). Most importantly, this thoracic osteoderm of UMNH VP 28351 exhibits a deep groove extending craniocaudally along the apex of the keel, as in WSC 16505.5. This “split-keel” morphology supports referral of UMNH VP 28351 to *Invictarx*.

The final thoracic morphotype is represented by numerous flat, thin osteoderm fragments. Most of these are simply nondescript shards; however, two examples are sufficiently large and well-preserved for comparison with other osteoderms. Both examples are quite thin, only 0.6 cm thick at their thickest preserved points. Their preserved margins are highly rugose (Figs. 10M–10O). On the larger of the two fragments, siderite infilling has highlighted the presence of abundant small pits of roughly uniform size and random distribution; the external surface texture is otherwise smooth (Fig. 10O). In all these features, these osteoderms of UMNH VP 28351 match WSC 16505.6, a thoracic interstitial ossicle in the holotype of *I. zephyri* (Figs. 4M–4R).

##### Pelvic osteoderms

The pelvic osteoderms of *I. zephyri* have been described earlier in this paper based upon two small fragments in UMNH VP 28350 (see Pelvic osteoderms of UMNH VP 28350 above), which show some features of Morphotype C in *G. mimus* (Burns, 2008), including flat external and basal surfaces and thick, non-tapering margins (Figs. 7V and 7W). UMNH VP 28351 provides additional information on the pelvic armor of *Invictarx* in the form of an incomplete osteoderm that strongly resembles the individual polygonal osteoderms that comprise the Category 3 co-ossified pelvic shields present in several nodosaurids (Arbour, Burns & Currie, 2011). The ankylosaurid *A. coombsi* also exhibits a Category 3 pelvic shield (Ford & Kirkland, 2001; Arbour & Currie, 2016); however, the pelvic osteoderms of *Aletopelta* are extremely thin (San Diego Natural History Museum (SDNHM) 33909), as is typical for ankylosaurid osteoderms (Burns & Currie, 2014).

**Figure 11:**
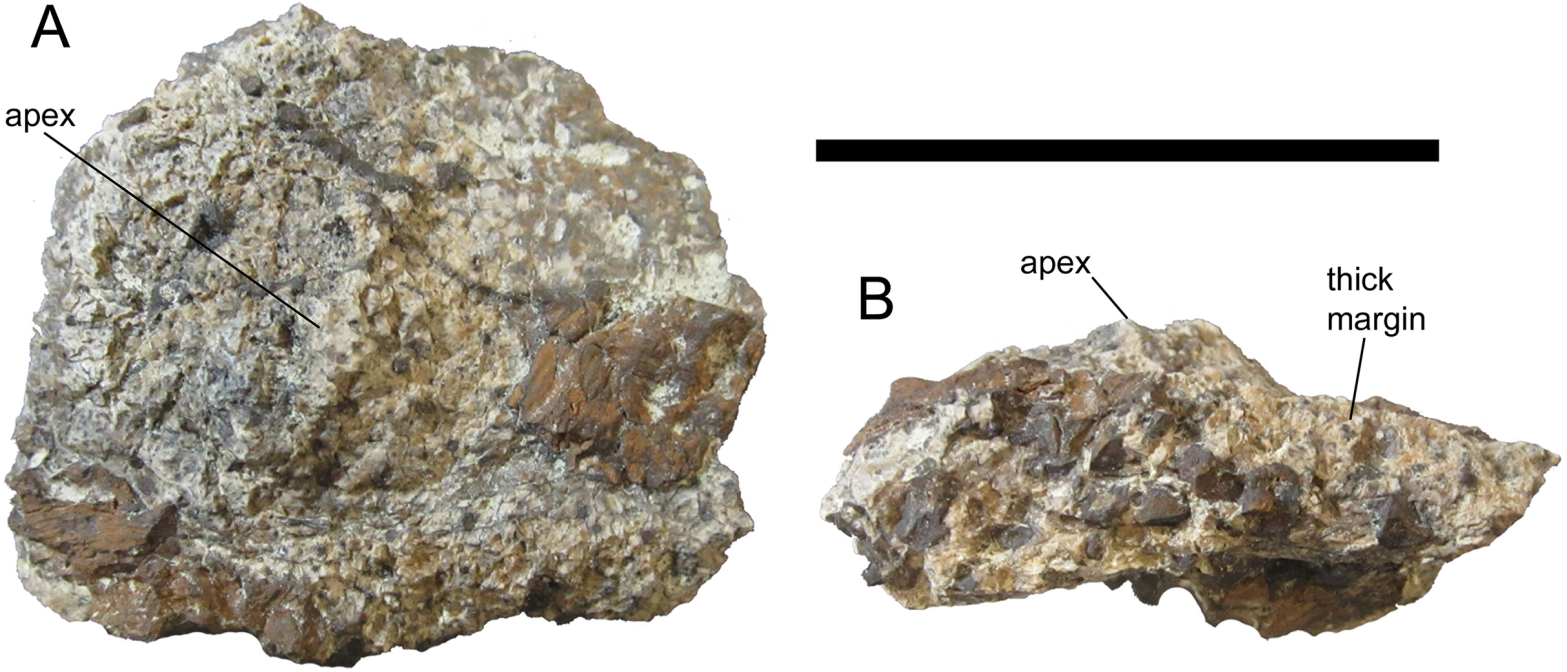
Pelvic osteoderm of UMNH VP 28351, referred specimen of *I. zephyri*. Pelvic osteoderm in (A) external and (B) marginal views. Scale bar equals five cm.

The pelvic osteoderm of UMNH VP 28351 bears a close resemblance to the osteoderms that form the Category 3 pelvic shield of the nodosaurid *G. mimus* (USNM 8610) (Morphotype C of Burns (2008)). The basal surface is flat. The external surface is mostly flat, including near the only preserved margin, except for a gentle, rounded apex (Fig. 11). The osteoderm does not become thinner toward its preserved margin, but rather remains very thick (1.2 cm at the margin’s thickest point). Based upon these similarities, it is likely that *I. zephyri* possessed a Category 3 pelvic shield consisting of co-ossified polygonal osteoderms of uniform or subequal size. Among other nodosaurids that have Category 3 pelvic shields, *G. mimus* (USNM 8610) (Burns, 2008) and *S. landerensis* (FMNH UR88) (Moodie, 1910; Carpenter & Kirkland, 1998) also exhibit gentle apices on the individual osteoderms. In *E. carbonensis* (Kirkland et al., 2013) and *N. textilis* (Lull, 1921), the individual osteoderms have completely flat external surfaces, lacking apices. The thickness of the pelvic osteoderm of UMNH VP 28351 was compared to a laser-scanned and 3D-printed replica of the pelvic shield of USNM 8610, the holotype of *Glyptodontopelta*. The pelvic shield of USNM 8610 includes five co-ossified nearly complete osteoderms (Fig. 1A in Burns (2008)). The thickest of these has an apicobasal thickness of 1.9 cm and a marginal thickness of 0.9 cm. The apicobasal thickness of the pelvic osteoderm of UMNH VP 28351 is 2.0 cm, and the maximum preserved marginal thickness is 1.2 cm, only slightly thicker than the osteoderm of USNM 8610.

## Discussion

Compared to many other nodosaurids, such as *Borealopelta*, *Europelta*, *Panoplosaurus*, *Edmontonia*, *Sauropelta*, and *Silvisaurus*, *Invictarx* is known from fragmentary remains. Nevertheless, it can be diagnosed by a unique combination of characters that unites its holotype and referred specimens, and distinguishes them from all other nodosaurid specimens. The morphology, stratigraphic positions, and geographical occurrence of the holotype and referred specimens are consistent with assignment to a single, new taxon. The status of *Invictarx* is similar to that of fellow nodosaurid *Glyptodontopelta*, which is known almost solely from osteoderms and is diagnosed by its unique osteoderm external surface texture and the morphology of its medial cervical osteoderms (Burns, 2008). Although *Glyptodontopelta* is represented by more specimens and a greater total number of osteoderms than *Invictarx*, the three specimens of *Invictarx* provide similar anatomical coverage to the known material of *Glyptodontopelta*, with osteoderms and ossicles from the cervical/pectoral, thoracic, and pelvic regions.

The definition of Nodosauridae proposed by Sereno (2005) is adopted in this paper: the most inclusive clade containing *P. mirus* and *N. textilis*, but not *Ankylosaurus magniventris. Invictarx*, from the early Campanian of New Mexico, is temporally situated between *Nodosaurus* and *Stegopelta* from the Cenomanian of Wyoming (Carpenter & Kirkland, 1998), and *Glyptodontopelta* from the early Maastrichtian of New Mexico (Burns, 2008; Jasinski, Sullivan & Lucas, 2011). This occurrence, plus the newly described nodosaurid *Acantholipan gonzalezi* from the Santonian of Coahuila, Mexico (Rivera-Sylva et al., 2018), indicates that nodosaurids persisted in Laramidia throughout the Late Cretaceous. In contrast, ankylosaurids suffered a local extinction in Laramidia concurrent with the inundation of the Western Interior Seaway in the Cenomanian and did not reinvade Laramidia until the Campanian (Arbour, Zanno & Gates, 2016). Further material and analysis will be necessary to explore the phylogenetic relationships and biogeographic significance of *Invictarx*.

## Conclusions

The new nodosaurid *I. zephyri* provides further insight into the poorly known vertebrate fossil record of the Allison Member of the Menefee Formation. Although the known material is fragmentary, the osteoderms exhibit a unique combination of characters. The occurrence of *Invictarx* in the early Campanian of southern Laramidia aligns with previous hypotheses that nodosaurids were present in Laramidia throughout the Late Cretaceous, even as ankylosaurids suffered a local extinction and later reinvaded from Asia (Arbour, Zanno & Gates, 2016).

## Supplemental Information

10.7717/peerj.5435/supp-1 Supplemental Information 1 Table S1. Table of Measurements of UMNH VP 28350. Measurements of select axial and appendicular elements of UMNH VP 28350, referred to *Invictarx zephyri* gen. et sp. nov.

## Institutional Abbreviations

AMNH: American Museum of Natural History, New York, New York, USA
FMNH: Field Museum of Natural History, Chicago, Illinois, USA
SDNHM: San Diego Natural History Museum, San Diego, California, USA
SMP: State Museum of Pennsylvania, Harrisburg, Pennsylvania, USA
TMP: Royal Tyrrell Museum of Palaeontology, Drumheller, Alberta, Canada
UMNH: Natural History Museum of Utah (formerly Utah Museum of Natural History), Salt Lake City, Utah, USA
USNM: National Museum of Natural History, Washington, DC, USA
WSC: Western Science Center, Hemet, California, USA.

## Additional Information and Declarations

### Field Study Permissions

The following information was supplied relating to field study approvals (i.e., approving body and any reference numbers): The fossil specimens described herein were collected under the following permits issued by the U.S. Bureau of Land Management: NM11-005S, NM12-03S, and NM16-11S.

### Data Availability

The following information was supplied regarding data availability: The data include measurements of one of the fossils. The fossil specimens described in the manuscript are accessioned at the Western Science Center (WSC) and Natural History Museum of Utah (UMNH) as WSC 16505, UMNH VP 28350, and UMNH VP 28351.

### New Species Registration

The following information was supplied regarding the registration of a newly described species: Publication LSID: urn:lsid:zoobank.org:pub:0E04AA40-BEA4-4A22-9CCB-6ACE419607B5. *Invictarx zephyri*; genus name LSID: urn:lsid:zoobank.org:act:C32ED4E1-3773-49A8-A314-68813708DF0F; Species name LSID: urn:lsid:zoobank.org:act:223160EC-F6B7-4637-9C2F-796EAF4A2816.
