# Supplementary material for: A new nodosaurid ankylosaur (Dinosauria: Thyreophora) from the Upper Cretaceous Menefee Formation of New Mexico
Source: PeerJ. 2018 Aug 24;6:e5435. doi: 10.7717/peerj.5435 (PMC6110256; doi:10.7717/peerj.5435)
Supplement: Supplemental Information 1 — Measurements of select axial and appendicular elements of UMNH VP 28350, referred to Invictarx zephyri gen. et sp. nov. [file peerj-06-5435-s001.docx]

**S1 Table. Table of Measurements of UMNH VP 28350.** Measurements of select axial and appendicular elements of UMNH VP 28350, referred to *Invictarx zephyri* gen. et sp. nov.

| **Elements** | **Measurements (in centimeters)** |
| --- | --- |
| Middle dorsal vertebra in Fig. 5A–D: |  |
| Craniocaudal length of centrum | 7.5 |
| Dorsoventral height of cranial centrum face | 6.3 |
| Width of cranial centrum face | 6.9 |
| Dorsoventral height of caudal centrum face | 7.0 |
| Width of caudal centrum face | 7.3 |
|  |  |
| Middle dorsal vertebra in Fig. 5E–H: |  |
| Craniocaudal length of centrum | 6.4 |
| Dorsoventral height of cranial centrum face | 6.0 |
| Width of cranial centrum face | 6.2 |
| Dorsoventral height of caudal centrum face | 6.0 |
| Width of caudal centrum face | 6.3 |
|  |  |
| Middle dorsal vertebra in Fig. 5I–L: |  |
| Craniocaudal length of centrum | 6.8 |
| Dorsoventral height of cranial centrum face | 6.1 |
| Width of cranial centrum face | 4.6 |
| Dorsoventral height of caudal centrum face | 6.2 |
| Width of caudal centrum face | 4.7 |
|  |  |
| Distal right humerus: |  |
| Mediolateral width of distal end | 13.7 |
|  |  |
| Proximal right radius: |  |
| Maximum preserved diameter of proximal end | 7.5 |
